# Stress associated gene expression in blood cells is related to outcome in radiotherapy treated head and neck cancer patients

**DOI:** 10.1186/1471-2407-12-426

**Published:** 2012-09-25

**Authors:** Siv K Bøhn, Kjell M Russnes, Amrit K Sakhi, Magne Thoresen, Marit Holden, JanØ Moskaug, Mari C Myhrstad, Ole K Olstad, Sigbjørn Smeland, Rune Blomhoff

**Affiliations:** 1 Department of Nutrition, Institute of Basic Medical Sciences, University of Oslo, Oslo, 0316, Norway; 2 Division of Cancer, Surgery and Transplantation, Oslo University Hospital, Oslo, 0310, Norway; 3 Department of Biostatistics, Institute of Basic Medical Sciences, University of Oslo, Oslo, 0316, Norway; 4 Norwegian Computing Center, Oslo, 0314, Norway; 5 Department of Biochemistry, Institute of Basic Medical Sciences, University of Oslo, Oslo, 0316, Norway; 6 Department of Clinical Chemistry, Oslo University Hospital, Ullevål, Oslo, 0407, Norway; 7 Institute for Clinical Medicine, University of Oslo, Oslo, 0316, Norway

**Keywords:** Radiotherapy, HNSCC, Antioxidants, Microarray, GSEA, Cancer

## Abstract

**Background:**

We previously observed that a radiotherapy-induced biochemical response in plasma was associated with favourable outcome in head and neck squamous carcinoma cancer (HNSCC) patients. The aim of the present study was to compare stress associated blood cell gene expression between two sub-groups of HNSCC patients with different biochemical responses to radiotherapy.

**Methods:**

Out of 87 patients (histologically verified), 10 biochemical ‘responders’ having a high relative increase in plasma oxidative damage and a concomitant decrease in plasma antioxidants during radiotherapy and 10 ‘poor-responders’ were selected for gene-expression analysis and compared using gene set enrichment analysis.

**Results:**

There was a significant induction of stress-relevant gene-sets in the responders following radiotherapy compared to the poor-responders. The relevance of the involvement of similar stress associated gene expression for HNSCC cancer and radioresistance was verified using two publicly available data sets of 42 HNSCC cases and 14 controls (GEO GSE6791), and radiation resistant and radiation sensitive HNSCC xenografts (E-GEOD-9716).

**Conclusions:**

Radiotherapy induces a systemic stress response, as revealed by induction of stress relevant gene expression in blood cells, which is associated to favourable outcome in a cohort of 87 HNSCC patients. Whether these changes in gene expression reflects a systemic effect or are biomarkers of the tumour micro-environmental status needs further study.

**Trial registration:**

Raw data are available at ArrayExpress under accession number E-MEXP-2460.

## Background

The strategy of cancer radiotherapy (RT) involves the eradication of cancer cells while sparing the surrounding normal tissues. When cells are exposed to RT, stress responses leading to the apoptotic cell death of tumour cells are activated. The global tissue responses to RT seem to be directed towards limiting damage, inducing repair processes and restoring tissue homeostasis [1]. However, it is not known how the systemic response to RT affects outcome.

The main risk factors associated with head and neck squamous carcinoma cancer (HNSCC) are related to an unhealthy lifestyle accompanied by alcohol and tobacco use and low daily intake of fruits and vegetables [2,3]. According to the second expert panel report on food and cancer prevention, non-starchy vegetables, fruits, and also foods containing carotenoids probably protect against the development of HNSCC [4]. We have previously observed in a pilot study that high levels of both post-RT carotenoids (biomarkers of fruit and vegetable intake) and endogenous plasma antioxidants (glutathione [GSH]) show a significant positive association with survival in patients with HNSCC [5,6]. In a larger follow-up study with 87 HNSCC patients and 100 healthy controls the effect of RT on a variety of biomarkers were assessed and pre-RT levels were compared with those in healthy controls [7]. Dietary antioxidants (carotenoids, tocopherols and ascorbic acid) and ferric reducing antioxidant power (FRAP) were lower in HNSCC patients compared to controls and dietary antioxidants decreased during RT. High pre-RT plasma antioxidant levels were positively associated with survival. During RT, decrease in ferric reducing power analysis (FRAP) was positively associated to survival. Similarly, high RT-induced increase in total hydryoperoxides (derivatives of reactive oxygen species [DROM]; plasma biomarker of oxidative stress) was also positively associated with survival [7].

Based on these results we selected one subgroup of patients that had responded to RT with a high increase in DROM and a concomitant decrease in FRAP (responders) and a subgroup of patients with the inverse combination of responses (poor-responders). Changes in gene expression is a major component of stress responses [8]. The primary aim of our study was therefore to compare these subgroups to identify differential blood cell gene expression relevant for stress and defence responses, such as DNA repair and apoptosis, both before (pre-RT) and during RT treatment. Such responses have previously been reported to be implicated in tumour development and radiation resistance in human cancers [9]. The relevance of the involvement of similar stress-associated gene-expression for HNSCC cancer and radio-resistance was therefore verified using two independent publicly available gene expression datasets.

## Methods

### Study population, sample preparation and analysis

This study reports the results of a subgroup of a larger study of HNSCC patients recruited from the Division of Cancer Medicine and Radiotherapy, Norwegian Radium Hospital, Rikshospitalet University Hospital in the period from May 2003 to May 2006 [7]. All the patients gave their written informed consent and the study was approved by the Regional Committee for Medical Research Ethics. The patients received either post-operative RT or RT alone for a period of 5–7 weeks. Radiation doses ranged from 50 Gy to 70 Gy. The inclusion criteria and patient characteristics as well as methods for sample preparation and plasma biomarker analyses are described by Sakhi et al. [7].

Samples for whole blood RNA isolation were obtained before and after RT using PAXgene Blood RNA Tubes (QIAGEN, Cat. No. 762115). PAX tubes were stored for 3 days at 4°C before RNA isolation and maintained at -80°C until microarray analysis.

### RNA isolation and microarray

Whole blood RNA was isolated according to the method detailed in the PAX kit handbook including the optional on-column DNase digestion. All samples (n = 2x20) had good RNA integrity as judged by a Bioanalyzer and sufficient yield (>5 μg RNA). Affymetrix, one-cycle gene expression protocol was performed including the globin transcription reduction (GeneChip®Globin-Reduction: Affymetrix) step. All reagents were purchased from Affymetrix.

Fragmented and biotinylated cRNA was hybridised to the Affymetrix Human Genome U133 Plus 2.0 arrays according to Affymetrix protocols. Scanning of arrays and image analysis were performed using GeneChip ® Scanner 3000 7 G and Operating Software 1.4 from Affymetrix.

Affymetrix HG-U133 microarray data for the radioresistant and radiosensitive human tumour xenografts and the data set of 42 head and neck squamous cell carcinoma cases and 14 controls were downloaded from Array-Express, (accession number E-GEOD-9716 and GEO, GSE6791 respectively) where the description of RNA isolation, hybridization and the labelling protocol is registered. The following raw data files were used from E-GEOD-9716: GSM245389, GSM245390, GSM245391, GSM245395, GSM245396 and GSM245397. Results using this dataset have been published by Khodarev et al. [10].

### Data analysis

The MADMAX quality control (QC) pipeline (https://madmax.bioinformatics.nl) was applied separately for the clinical trial data and the human tumour xenograft data to assess array quality and for robust multiarray average (RMA)-normalisation. Two arrays from the clinical trial failed QC criteria and were thus discarded. Baseline comparisons in both the clinical trial and xenografts were performed on log-transformed data (base 2). Changes in gene expression during RT were obtained by calculating log2 ratios between the before and after RT intensities for every gene after RMA normalisation. The groups were then compared with regard to this ratio. Probe set annotation was updated via the NetAffx on the Affymetrix website. The annotation file was last updated on March 12^th^ 2009. MIAME standards [11] were followed in the analysis and storage of data. The raw data are available at ArrayExpress by accession number E-MEXP-2460 http://www.ebi.ac.uk/microarray-as/ae/.

### Identification of significantly differentially expressed genes at baseline and during RT

BAMarray^TM^, a Bayesian ANOVA method for the analysis of microarray data that adjusts for multiple testing, was used to identify differentially regulated gene transcripts [12].

### Gene set enrichment analysis (GSEA)

Gene set enrichment analysis GSEA [13] was used to test whether groups of genes involved in stress and defence processes were differentially expressed pre-RT or differentially changed during RT by comparing the ‘responders’ with the ‘poor-responders’ using J-express (http://www.molmine.com). Gene set collections associated with stress functions were obtained using a gene set browser (Molecular Signatures Database v3.0) on the Broad institute website (http://www.broad.mit.edu/gsea/), as previously described [14] using the keywords such as ‘apoptosis’, ‘hypoxi*’ and others. The large, predefined gene set collection; C3 (TFT), was also tested. In C3 (TFT) the genes are grouped if they share a transcription factor binding site (http://www.broad.mit.edu/gsea/). FDR q-values < 5% was considered statistically significant.

### Data analysis of biomarkers

SPSS (version 14.0) was employed to compare the pre-RT levels and changes in FRAP, DROM, ascorbic acid, tocopherols and the carotenoids. All comparisons were performed using the Mann–Whitney (MW) test considering P-values < 0.05 as being statistically significant. For clinical outcome overall survival was calculated from the start of RT until death by any cause or last follow-up examination. Progression-free survival was calculated from the start of RT until relapse of last follow-up examination. The number of survivors after 3 years was compared between the groups using the Fisher exact test. The survival distributions of the two groups were compared using the Log Rank test. Due to different practical limitations, appropriate sample collection was not possible for every analysis in all patients and controls. Thus, the numbers of samples were 15 for Glutathione disulfide (GSSG) and redox potential and 19 for total and reduced glutathione (GSH).

Principal component analysis (PCA) was used to summarise the main differences between the individuals using the Unscrambler Version 9.8 (http://www.camo.com). Detailed description of the analysis and how to interpret the results can be found in online Additional file 1. The PCA figures were optimized (colors, fonts) using Adobe illustrator CS2.

## Results

### Patient characteristics

**Figure 1:**
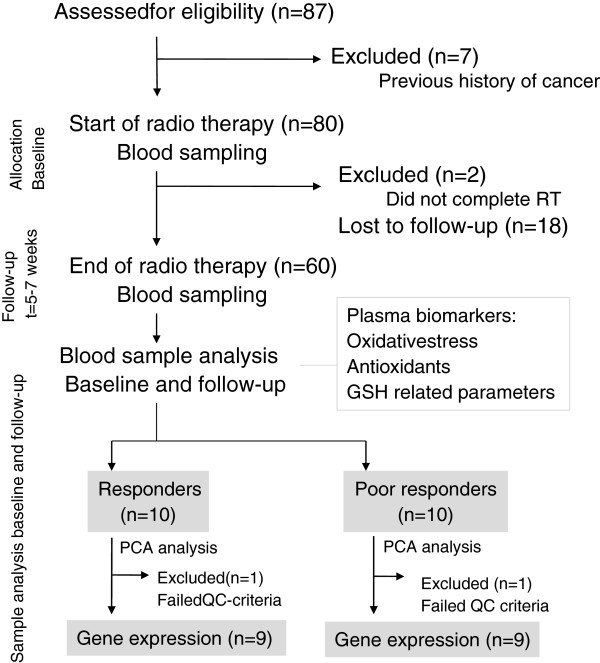
**CONSORT (Consolidated Standards Of Reporting Trials) diagram detailing the study.** ‘Responders’: high relative increase in DROM and concomitant decrease in FRAP in response to RT. ‘Poor responders’: low relative increase or decrease in DROM and concomitant low decrease or increase in FRAP in response to RT.

Out of the 87 patients with histologically verified HNSCC recruited in the present study, 10 biochemical ‘responders ’ and 10 ‘poor-responders’ were selected for gene expression analysis (Figure 1) according to the following inclusion criteria. Among the patients with a high (above median) negative change in plasma FRAP and a high positive change in DROM during RT, 10 samples were assigned to the responders group. Among the patients with low negative change in plasma FRAP and a low positive change in DROM during RT, 10 samples were assigned to the ‘poor-responders’ group.

The ‘responders’ were compared with the ‘poor responders’ with regard to baseline characteristics as summarised in Table 1. There were no statistically significant differences in baseline characteristics between the two groups except there were differences in outcome which was expected owing to the selection criteria’s.

**Table 1:** Baseline charachteristics of the responders and the poor responders

		**Poor responders**		**Responders**		**p**
	BMI median (25%, 75% percentiles)	22	(21,27)	25	(23,29)	0.32
	AGE (years, median (25%, 75% percentiles))	61	(55,64)	59	(55,76)	0.91
**Gender**	Men	9		10		
	Women	1		0		1.00^§^
**Localisation**	larynx	1		5		0.18^§^
	hypopharynx	3		0		
	oral cavity	4		3		
	oropharynx	2		2		
**Treatment**	RT	7		7		1.00^§^
	Surgery and RT	3		3		
**Stage**	I	0		1		0.34^§^
	II	1		3		
	III	1		2		
	IV	8		4		
**Smoking status**	Non smokers and former smokers	5		7		0.65^§^
	Smokers	5		3		
**Survival (3 Year)**	Median (months) (25%, 75% percentiles)	18	(11,25)	30	(19,40)	<0.01
	Overall survivors (n)	4		10		0.01^§^
	Median time to relapse, (months) (25%, 75% percentiles)	6	(5,21)	26	(16,34)	<0.01
	Progression-free survivors (n)	2		8		0.02^§^

BMI and age were compared between the groups using the Mann–Whitney test. Median values are given with interquantile range in brackets. Gender, localization, treatment, stage, smoking status, number of overall survors and number of progression free survivors were compared using Fisher’s exact test(§). Survival was compared using the log-rank test.

### Effect of radiotherapy on whole blood stress associated gene-expression

GSEA [13] was used to test whether gene sets associated to stress responses were differentially changed during RT in the ‘responders’ compared with the ‘poor responders’. The stress associated gene sets were, in general, significantly upregulated in the ‘responders’ relative to the ‘poor-responders’ (Table 2). These results indicate that the group with the best prognosis had a significantly higher induced stress-associated gene expression in response to RT. Lists of the gene sets that were differentially changed in ‘responders’ and ‘poor-responders’ can be explored in Additional file 2: Table S2 and Additional file 3: Table S3. used to test if gene sets associated with stress responses were differentially expressed at pre-RT in the 'responders' and ‘poor responders’. Table 2 lists the number of gene sets from each of the tested gene set collections that were significantly different between the groups’ pre-RT and the number of gene sets differentially changed during RT (FDR < 5%). In the ‘poor-responders’ a substantial amount of ‘stress’ gene sets were significantly higher expressed at pre-RT when compared to the‘ responders’ (FDR < 5%). A list of the differentially expressed gene sets between the ‘responders’ and ‘poor-responders’ at pre-RT can be viewed in Additional file 4: Table S4 and Additional file 5: Table S5.

**Table 2:** Summary of GSEA analysis comparing whole blood gene expression profiles of poor-responders (PR) to responders (R) with regard to pre-RT gene expression levels and response to RT

**Stress and defence relevant gene set database (keyword)**	**Number of gene sets in collection**	**HNSCC patients whole blood**			
	**HG-U133-plus2**	**Baseline expression pre-RT**		**Change during RT**	
		**PR > R**	**R > PR**	**PR > R**	**R > PR**
Apoptosis	273	29	0	0	91
Cell and cycle	311	38	1	0	85
Cytokine*	239	41	0	0	89
Hypoxi*	63	5	0	0	25
Antioxidant*	10	8	0	0	4
Immune* and response	116	10	0	0	37
Stress and response	59	5	0	0	11
DNA and repair	80	12	0	0	13
Cancer	699	75	1	4	152
Tox*	136	23	0	1	49
C3 TFT	583	18	11	0	4

The number of stress and defence-relevant gene sets that were higher expressed in one group compared to the other are given for each gene set collection. FDR (false discovery rate) < 5%.

Affymetrix HG-U133-plus2 chips were used to analyze the whole blood RNA. A gene set browser provided by Broad institute at www.broad.mit.edu/gsea/ was used to obtain the stress-associated gene set collections. Detailed information regarding the regulated gene sets is given in online Additional files 2-5. *indicates that truncated search keywords were used to create the gene set collections. C3TFT is a pre-defined gene set collections.

Most of the gene sets that were higher expressed pre-RT in the ‘poor-responders’ when compared to ‘responders’ were also found in the list of gene sets that were induced significantly in the ‘responders’ when compared to ‘poor-responders’ (FDR < 5%). However, the number of stress-associated gene-sets significantly induced in the‘ responders’ during RT was however much higher than the number of gene-sets that were higher expressed at pre-RT in the ‘non-responders’.

Furthermore we observed 4 significantly upregulated C3TFT gene sets in the ‘responders’ group as compared to the ‘poor-responders’ (FDR < 5%). Two of the up-regulated gene sets had regulatory motifs for unknown transcription factors and 2 gene sets have motifs for the known transcription factors CCAAT/enhancer binding protein (CEBPB) and nuclear respiratory factor 2 (Additional file 2: Table S2). Both transcription factors are involved in stress and defence responses [15,16].

### Relevance of stress associated gene expression for radio-resistance

GSEA on a publicly available data set of radiation resistant versus radiation sensitive HNSCC xenografts (E-GEOD-9716) revealed that the radioresistant tumour-xenografts had a higher basal expression of stress related gene sets (GSEA, FDR < 5%) as compared with the radio-sensitive xenografts (Table 3). All C3TFT gene-sets that were higher expressed in the radioresistant xenografts, were associated with interferon signalling. Lists of the regulated gene sets can be explored in online Additional file 6: Tables S6 and Additional file 7: Table S7.

**Table 3:** Summary of GSEA analysis comparing radioresistant (RR) with radiosensitive (RS) HNSCC xenografts in nude mice and HNSCC tumour tissue versus normal epithelial cells

**Stress and defence relevant gene set database (keyword)**	**Number of gene sets in collection**		**HNSCC tumour cell xenografts (GEOD-9716) Baseline pre-RT**		**HNSCC tumour versus controls (GEOGSE6791) Baseline pre-RT**	
	**HG-U133-plus2**	**HG-U133A**	**RR> RS**	**RS>RR**	**HNSCC>ctr**	**ctr>HNSCC**
Apoptosis	271	272	30	1	163	1
Cell and cycle	308	309	63	1	200	2
Cytokine*	239	236	33	0	104	2
Hypoxi*	63	63	7	0	41	0
Antioxidant*	10	10	2	0	2	0
Immune* and response	114	114	23	0	55	1
Stress and response	59	58	6	0	0	0
DNA and repair	80	79	14	0	57	0
Cancer	699	687	103	14	373	9
Tox*	137	140	16	2	70	2
C3 TFT	582	587	5	2	83	1

The number of stress and defence-relevant gene sets that were more highly expressed in one group relative to the other is given for each gene set collection. False discovery rate (FDR) <5%.

Affymetrix HG-U133A microarray chips were used in the GEOD-9716 dataset while Affymetrix HG-U133-plus2 chips were used in the GSE6791 dataset. A gene set browser provided by the Broad institute at www.broad.mit.edu/gsea/ was used to obtain the stress-associated gene set collections. Detailed information regarding the regulated gene sets is given in online Additional files 6-9. *indicates that truncated search keywords were used to create the gene set collections. C3TFT is a pre-defined gene set collection.

### Relevance of stress associated gene expression for HNSCC

GSEA on a publicly available data set of 42 head and neck squamous cell carcinoma cases and 14 controls (GEO, GSE6791), revealed that HNSCC tumour tissue had a higher basal expression of stress related gene sets (GSEA, FDR < 5%) compared to normal endothelial cells from healthy controls (Table 3). The C3TFT gene-sets that were more highlyexpressed in the tumour tissue had common regulatory motives for various E2Fs, NRF2, ARNT, NRF1, MYC and YY1. Lists of the regulated gene sets can be explored in online Additional file 8: Table S8 and Additional file 9: Table S9.

### Identification of significantly differentially expressed genes before RT and during RT comparing ‘responders’ with ‘poor-responders’

Another statistical method, BAM array^TM^[12], was used to identify significantly differentially expressed genes before and during RT. Pre-RT, 123 genes were significantly higher expressed in the ‘poor responders’ as compared to the ‘responders’ while 352 genes were more highly expressed in the ‘responders’ (Additional file 10: Table S10).

**Figure 2:**
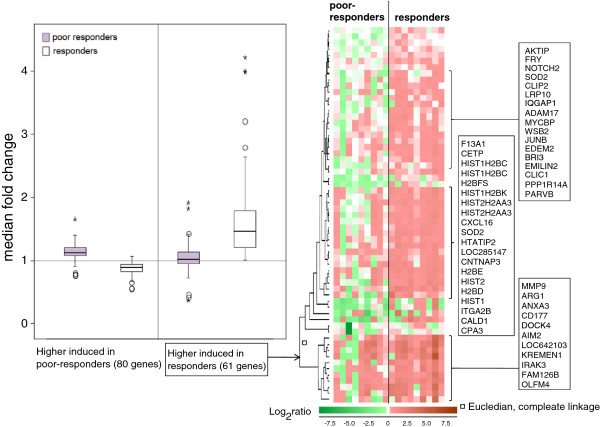
**Box plot of the median fold-change in the genes that were significantly differentially changed between the ‘poor responders’ and the ‘responders’ during RT.** The heatmap illustrates the 61 genes significantly induced by RT in the ‘responders’ as compared with ’non-responders’ listing the genes in the most up-regulated clusters.

During the RT period 61 gene transcripts were significantly induced in the ‘responders’ while 80 were higher induced in the ‘poor-responders’ (Additional file 11: Table S11). The median fold change for each significantly changed gene was calculated, and box plots were obtained to summarise the RT effects in ‘responders’ and ‘poor responders’ (Figure 2). The median fold change in gene-expression was higher in the ‘responders’ than in the ‘poor responders’. Among the gene transcripts significantly induced in the ‘responders’ (heatmap, Figure 2) several have previously been reported in association to RTnamely. CXCL16 and ADAM17 [17], JUNB [18], NOTCH [19], HTATIP2 [20,21], BRI3 [22], SOD2 [23] and MMP9 [24]. In addition eight histone variants of the H2B and H2A family [25] were significantly induced in the ‘responders’.

### Plasma biomarkers

Pre-RT plasma biomarkers and changes in these biomarkers during RT are summarised in Additional file 12, Table S12 presented as median values with the corresponding 95%CI. Principal component analysis (PCA) was used to summarise the main differences between the groups with regard to plasma biomarkers and information on staging of tumour. Principal component (PC) 1 and PC 2 separated the ‘responders’ from the ‘poor-responders’ with regard to pre-RT biomarkers and RT-induced changes (Additional file 1: Figure S1A-D). The Additional file 1 includes a detailed description of how the figures are interpreted and the results. Additional file 1: Figure S1A, correlation loading plot, shows that the pre-RT plasma carotenoids (lycopene, zeaxanthin, lutein, α-carotene and β-carotene) and ascorbic acid (AA) correlate, and are associated to the ‘responders’.

On the other hand pre-RT DROM shows an inverse correlation to the carotenoids and is associated to the ‘poor-responders’. Thus the ‘responders’ had higher plasma levels of antioxidants and a lower level of oxidative stress biomarker (DROM) before RT than the ‘poor-responders’. Tumour stage seems not to be important in relation to the separation of those subgroups.

The correlation loading plot of Additional file 1: Figure S1D shows that the RT-induced changes in antioxidants (carotenoids and ascorbic acid) are important for PC1 and therefore explains the group effect. The changes in plasma antioxidants are highly correlated and the negative change in plasma antioxidants, increased ratio of oxidised vitamin C/ vitamin C and increased DROM are associated with the ‘responders’. Thus the ‘responders’ had higher induced levels of oxidative stress biomarkers (oxidised vitamin C/ vitamin C and DROM) during RT than the ‘poor-responders’ while using more plasma antioxidants.

## Discussion

The cornerstone of RT for cancer treatment is the induction of the oxidative stress with the purpose of killing the tumour while sparing the normal tissue. To our knowledge, it remains unknown as to how the host response (i.e. the systemic response) to treatment affects the outcome. We found that induction of systemic stress responses seems to be involved in the successful response to radiation therapy in HNSCC patients.

The results of this study is based on comparison of two sub-groups of a previously published study [7]. In this study Sakhi et al. demonstrated that a decrease in FRAP during RT was positively associated to survival and that a high RT-induced increase in plasma biomarker of oxidative stress (DROM) was similarly positively associated with survival. Based on these results we identified two subgroups of HNSCC patients with different response-signatures to RT with regard to changes in oxidative stress biomarkers (DROM), plasma antioxidants (FRAP) and outcome. The patients who responded to RT with a high increase in DROM and concomitant decrease in FRAP (the ‘responders’) were compared with the patients who responded with the inverse combination of these two parameters (the ‘poor responders’). We identified different responses to RT with regard to modulation of gene-expression in whole blood by means of whole blood gene expression. The responders exhibited a significantly higher induction of genes associated with stress responses such as DNA repair, apoptosis, and hypoxia and also genes relevant to immune response. Furthermore, the responders had a higher consumption of plasma antioxidants indicated by decreased plasma carotenoids, vitamin C and FRAP.

We also found that the ‘poor-responders’ with the poor outcome had a significantly higher pre-RT stress status as revealed by their gene expression profiles and also plasma biomarkers (DROM and ratio of oxidized/reduced AA). The poor responders also had a significantly lower levelof plasma carotenoids pre-RT. We have previously suggested that a study designed to test whether increased intake of fruit and vegetables before start of RT can improve survival would be valuable [6,7]. The current observation that patients with a high pre-RT stress associated gene expression also had lower levels of fruit and vegetable biomarkers supports this suggestion. Most likely, plant foods may not only protect against oxidative stress and the subsequent oxidative damage due to antioxidant abilities but also activate adaptive defence mechanisms [14,26].

One may speculate that the observed systemic stress responses measured in blood cells are biomarkers of changes in host characteristics during RT and therefore potentially linked to modifiable lifestyle factors as for instance diet or antioxidant intake. Treatment response in oncology is not only dependent of tumour characteristics and the therapy given, but also by host factors such as the tumour microenvironment (including fibroblasts, endothelial cells and immune cells) [27]. In addition, oxidative stress status in the tumour microenvironment is possibly important for tumour progression [28].

The lack of treatment response in the ‘poor-responders’ might be related to a higher initial pre-RT stress level. A high pre-RT stress level could indicate that the stress resistance mechanisms in the poor responders are chronically high and thereby prohibit an adequate response to RT. Increased defence mechanisms in tumour cells have been associated with RT resistance both *in vitro* and *in vivo.* For example; it is known that radiation sensitivity is related to the efficiency of DNA double-strand break repair. Defects/loss of function in genes involved in DNA repair can thus enhance radiation sensitivity. Inhibition of other stress protective proteins, such as the Hsp90, also enhances the radiosensitivity both *in vitro* and in HNSCC xenograft models [29].

Interestingly we verified that a similar stress relevant gene expression pattern was significantly higher expressed in tumour tissue compared to normal epithelial cells in an independent publicly available data set of HNSCC patients and normal controls.

It is also likely that the stress associated gene expression pattern is involved in RT resistance mechanisms. Overexpression of stress relevant proteins such as GSH-related enzymes and HIF1α in tumours has been shown to participate in oncogenesis and in resistance to both RT and chemotherapy [30-33]. Increased expression of endogenous antioxidants has also been hypothesized to be at least partially responsible for radiation-induced adaptive responses [34-38]. We therefore tested whether similar stress-associated gene-expression profile could be relevant for RT resistance and used a publicly available gene expression dataset of radioresistant and radiosensitive xenografts for this purpose. Stress associated gene expression was found to be relevant for radioresistance. Our results are in line with the main findings from the source publication for the xenograft dataset reporting overexpression of IFN/STAT signalling in the radioresistant xenografts [10]. Several of the gene sets that were more highly expressed in the radioresistant xenografts overlapped with those that were found to be differentially expressed between the ‘responders’ and ‘poor responders’ both before and during RT.

Although there was no statistical difference in patients’ characteristics between the two groups, it can be argued that the cohort is not well balanced with regard to site of origin, staging and the low number of samples used. In particular there were 3 hypopharynx cases in the poor responders and 0 cases in the responders which potentially could have an impact on the results since patients with this tumour subsite have a worse outcome than patients with other tumour localizations [39]. Consequently we repeated the GSEA analysis on a dataset that excluded the hypopharynx cases and showed that it did not affect the results noteworthy (data not shown). In addition, because stage of disease is associated with outcome in HNSCC patients [7] we included tumour stage as a parameter in the PCA analysis. Stage does not seem to be important for the different biochemical response to RT for the two groups in our study.

Although we have identified a biomarker panel that is associated with outcome in patients that received RT we cannot exclude that the changes that are induced during RT period could have been induced or affected by other stress factors during treatment (i.e. surgery, changes in nutrition, weight loss, fungal infections and other factors).

## Conclusion

Although RT is a locoregional treatment modality, we found systemic changes in the gene expression in non-tumour cells i.e. blood cells. We demonstrated that the induction of a systemic stress response, stress-relevant gene expression in blood, seems to be important for successful RT response and increased survival rates in HNSCC patients. Furthermore we used two publicly available data sets to validate that expression of stress associated genes is relevant for RT resistance and that tumour cells from HNSCC patients have a higher expression of these genes as compared with cells from healthy subjects.

Whether the observed changes in blood cell gene expression reflects a systemic effect or are biomarkers of the tumour microenvironment requires further elucidation.

## Abbreviations

ADAM: A disintegrin and metalloprotease domain; ARNT: Aryl hydrocarbon receptor nuclear translocator; BRI3: Brain protein I3; CEBPB: CCAAT/enhancer binding protein; CXCL: Chemokine (C-X-C motif) ligand; DHHA: Dehydroascorbic acid; DROM: Derivatives of reactive oxygen species; FRAP: Ferric reducing power analysis; GSEA: Gene set enrichment analysis; GSH: Glutathione; HNSCC: Head and neck squamous carcinoma cancer; HTATIP2: HIV-1 Tat interactive protein; IFN: Interferone; MMP9: Matrix metallopeptidase; MW: Mann–whitney; PCA: Principal component analysis; RMA: Robust multiarray average; RT: Radiotherapy; SOD: Superoxide dismutase; STAT: Signal-transducer and activator of transcription protein; YY1: Yin-yang transcription factor 1.

## Competing interests

The following authors declare no potential conflict of interest SKB, KMR, AKS, MT, MH, JØM, MCM, OKO and SS. RB has interests in Bioindex AS and Vitas AS. Bioindex was established by Birkeland Innovation, the technology transfer office at the University of Oslo while Vitas was established by Oslo Innovation Center.

## Authors information

Professor Sigbjørn Smeland is Head of The Cancer, Surgery and Transplantation Clinic at Oslo University Hospital which is one of the largest cancer clinics and cancer research institutes in Europe. Professor Rune Blomhoff is the Head of The Department of Nutrition at the University of Oslo, which is the largest nutrition unit in Europe that is associated with a medical faculty.

## Authors' contributions

SKB, drafted the manuscript, participated in formulation of the hypothesis and in the RNA-relevant work. She was responsible for the statistical analysis together with MT and MH. KMR, participated in formulation of the hypothesis, was responsible for sample collection and also responsible for acquisition of patient data together with AKS. AKS, participated in formulation of the hypothesis and was responsible for sample collection and acquisition of patient data together with KMR. She performed the biomarker analysis in patient samples. MT, participated in formulation of the hypothesis and was responsible for the statistical analysis together with MT and SKB. MH, was responsible for the statistical analysis together with SKB and MH. JØ. Moskaug participated in formulation of the hypothesis and participated in the RNA-relevant work. MCM participated in formulation of the hypothesis and in the RNA-relevant work. OKO carried out the array hybridisation. SS and RB participated in formulation of the hypothesis and revised the manuscript for important intellectual content. All authors revised and approved the final version before submission.

## Pre-publication history

The pre-publication history for this paper can be accessed here:

http://www.biomedcentral.com/1471-2407/12/426/prepub

## Supplementary Material

Additional file 1 **Figure S1.** Results from PCA analysis of plasma biomarkers including a description of the method used. Click here for file

Additional file 2 **Table S2.** List of gene sets that were more highly induced in responders compared to poor-responders during RT. Click here for file

Additional file 3 **Table S3.**List of gene sets that were more highly induced in poor-responders compared to responders during RT. Click here for file

Additional file 4 **Table S4.** List of gene sets that were more highly expressed in the ‘responders’ compared to ‘poor-responders’ at pre-RT. Click here for file

Additional file 5 **Table S5.** List of gene sets that were more highly expressed in poor-responders than in responders at pre-RT. Click here for file

Additional file 6 **Table S6.** List of gene sets that were more highly expressed in the radioresistant xenograft samples than in the radiation sensitive. Click here for file

Additional file 7 **Table S7.** List of gene sets that were more highly expressed in the radiosensitve xenograft samples than in the radioresistant. Click here for file

Additional file 8 **Table S8.** List of gene sets that were more highly expressed in HNSCC tumour tissue than innormal endothelial cells from controls. Click here for file

Additional file 9 **Table S9.** List of gene sets that were more highly expressed in normal endothelial cells from controls than in HNSCC tumour tissue. Click here for file

Additional file 10 **Table S10.** Genes significantly differentially expressed in responders compared to poor-responders at pre-RT (BAM array). Click here for file

Additional file 11 **Table S11.** Genes significantly differentially induced by RT in responders as compared with poor-responders. Click here for file

Additional file 12 **Table S12.** Summary of the pre-RT plasma biomarkers and RT induced changes in these biomarkers for responders and poor responders. Click here for file
