# Supplementary material for: Stress associated gene expression in blood cells is related to outcome in radiotherapy treated head and neck cancer patients
Source: BMC Cancer. 2012 Sep 25;12:426. doi: 10.1186/1471-2407-12-426 (PMC3517770; doi:10.1186/1471-2407-12-426)
Supplement: Additional file 1 — Figure S1. Results from PCA analysis of plasma biomarkers including a description of the method used. [file 1471-2407-12-426-S1.doc]

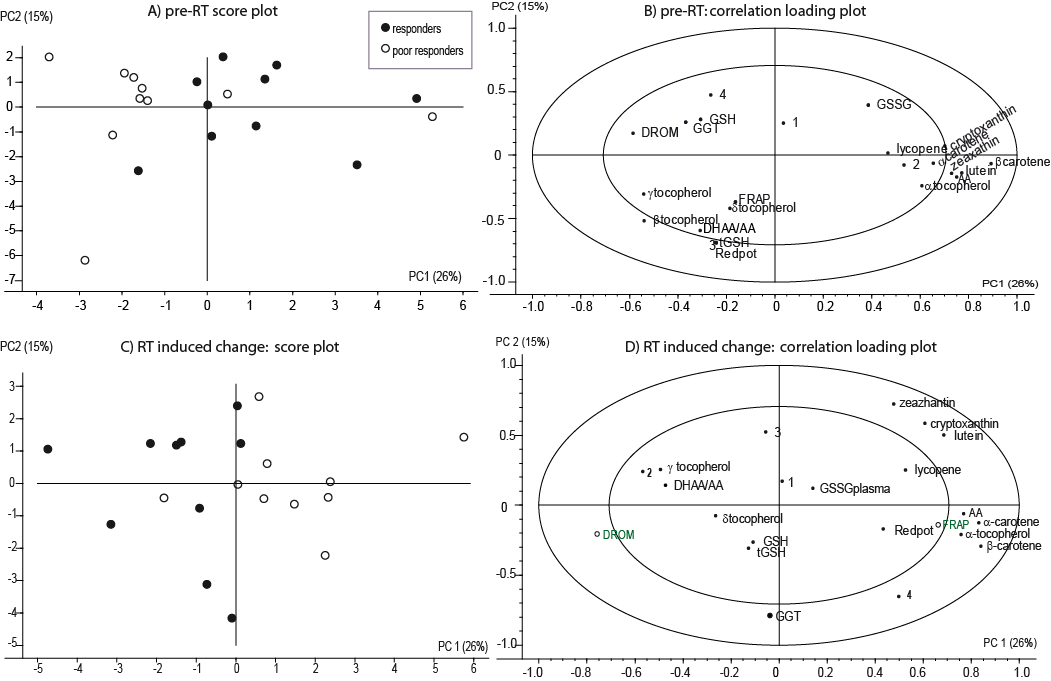
Bøhn et al, Supplementary file 11

Ascorbic acid (AA), DHAA; oxidized AA , GSH: glutathione (reduced) , GSSG: oxidized GSH, tGSH: total GSH, GGT: γ- glutamyl transferase, DROM: total hydroperoxides , FRAP: ferric reducing antioxidant power, Redpot; redox potential, 1-4= tumor stages. I-IV

PCA of pre-RT plasma variables (A and B) and RT induced changes (C and D). PCA scoring plots show separation of ’responders’ and ’poor-responders’ with regard to both pre-RT plasma variable levels (A) and RT-induced changes (C). The corresponding correlation loading plots (B and D) visualize the importance of the individual variables for the separation. FRAP and DROM (green) were passified in the PCA of RT-induced changes.

**Results (plasma biomarkers):**

PCA summarize the main differences between the groups with regard to plasma biomarkers and information on staging of tumor. Principal component (PC) 1 and PC 2 separate the ’responders’ from the ’poor-responders’ with regard to pre-RT biomarkers **(figure A and B)** and RT-induced changes **(figure C and D).** Figure A, correlation loading plot, shows that the pre-RT plasma carotenoids (lycopen, zeaxhantin, lutein, α-carotene, β-carotene) and ascorbic acid (AA) correlate, are important for PC 1 but not for PC2, and associated to the ’responders’.

Pre-RT DROM on the other hand shows an inverse correlation to the carotenoids and is associated to the ’poor-responders’. Thus the ’responders’ have higher plasma levels of antioxidants and less oxidative stress biomarker (DROM) before RT than the ’poor-responders’. The stageing of tumor seems not to be important for the separation of those subgroups.

Correlation loading plot of figure D shows that the RT-induced changes in antioxidants (carotenoids and ascorbic acid) are important for PC1 and therefore explains the group effect. The changes in plasma antioxidants are highly correlated and the negative change in plasma antioxidants, increased ratio of oxidized Vitamin C/ Vitamin C and increased DROM are associated to the ’responders’. Thus the ’responders’ have higher induced levels of oxidative stress biomarkers (oxidized Vitamin C/ Vitamin C and DROM) during RT than the ’poor-responders’ while using more plasma antioxidants.

In addition the responders had a significant increase the plasma levels of the inducible endogenous antioxidant enzyme, GSH. The post-RT GSH levels was also found to be significantly higher than in the ’poor responders’ which is in line with the results from a previous pilot study on a similar HNSCC patient group where high post-RT GSH levels were associated to survival [1].

Reference List

1. Bohn SK, Smeland S, Sakhi AK, Thoresen M, Russnes KM, Tausjo J, Svilaas A, Svilaas T, Blomhoff R: **Post-radiotherapy plasma total glutathione is associated to outcome in patients with head and neck squamous cell carcinoma.** *Cancer Lett* 2006, **238:**240-247.
